# Biochemical evidence that regulation of Ero1β activity in human cells does not involve the isoform-specific cysteine 262

**DOI:** 10.1042/BSR20130124

**Published:** 2014-03-31

**Authors:** Henning G. Hansen, Cecilie L. Søltoft, Jonas D. Schmidt, Julia Birk, Christian Appenzeller-Herzog, Lars Ellgaard

**Affiliations:** *Department of Biology, University of Copenhagen, 2200 Copenhagen, Denmark; †Department of Pharmaceutical Sciences, University of Basel, 4056 Basel, Switzerland

**Keywords:** disulfide-bond formation, endoplasmic reticulum oxidoreductin-1 (Ero1), redox regulation, unfolded protein response (UPR), AMS, 4-acetamido-4′-maleimidylstilbene-2,2′-disulfonic acid, ATF6α, activating transcription factor 6α, BiP, immunoglobulin heavy-chain-binding protein, DTT, dithiothreitol, Dox, doxycycline, ER, endoplasmic reticulum, Ero1, endoplasmic reticulum oxidoreductin-1, Ero1α-WT, wild-type Ero1α, HEK-293 cells, human embryonic kidney cells, HERP, homocysteine-induced endoplasmic reticulum (ER) protein, NEM, *N*-ethylmaleimide, PDI, protein disulfide-isomerase, PERK, PKR (double-stranded-RNA-dependent protein kinase)-like endoplasmic reticulum kinase, TCA, trichloroacetic acid, UPR, unfolded protein response

## Abstract

In the ER (endoplasmic reticulum) of human cells, disulfide bonds are predominantly generated by the two isoforms of Ero1 (ER oxidoreductin-1): Ero1α and Ero1β. The activity of Ero1α is tightly regulated through the formation of intramolecular disulfide bonds to help ensure balanced ER redox conditions. Ero1β is less tightly regulated, but the molecular details underlying control of activity are not as well characterized as for Ero1α. Ero1β contains an additional cysteine residue (Cys^262^), which has been suggested to engage in an isoform-specific regulatory disulfide bond with Cys^100^. However, we show that the two regulatory disulfide bonds in Ero1α are likely conserved in Ero1β (Cys^90^–Cys^130^ and Cys^95^–Cys^100^). Molecular modelling of the Ero1β structure predicted that the side chain of Cys^262^ is completely buried. Indeed, we found this cysteine to be reduced and partially protected from alkylation in the ER of living cells. Furthermore, mutation of Cys^100^–but not of Cys^262^–rendered Ero1β hyperactive in cells, as did mutation of Cys^130^. Ero1β hyperactivity induced the UPR (unfolded protein response) and resulted in oxidative perturbation of the ER redox state. We propose that features other than a distinct pattern of regulatory disulfide bonds determine the loose redox regulation of Ero1β relative to Ero1α.

## Abstract 2

Our findings indicate that the regulatory disulfide bonds are conserved in the human oxidases Ero1α and Ero1β. We therefore propose that features other than a distinct pattern of disulfide bonds determine the previously established difference in regulation of Ero1α and Ero1β activity.

## INTRODUCTION

**Figure 1:**
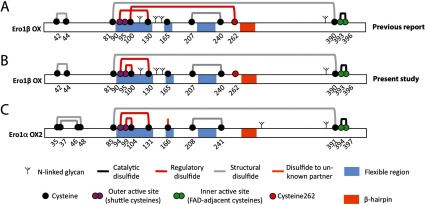
Disulfide bonds in Ero1β and Ero1α (**A**) Schematic representation of the proposed disulfide pattern in the OX redox form of Ero1β as reported by Wang et al. [7]. (**B**) Proposed disulfide bond pattern in Ero1β based on the present study. (**C**) Disulfide bond pattern in Ero1α OX2 verified by mass spectrometry [23,24] and crystallography [4]. The cysteine residues are shown in black, magenta (outer active site), green (inner active site) and red (Cys^262^ in Ero1β) with amino acid numbering. Disulfide bonds are depicted as thick grey (likely structural), black (active site) or red (reported regulatory function; Ero1β [7,32] and this study, Ero1α [4,23,24,47]) lines. The thick orange line at Cys^166^ indicates the connection to a potential (but unidentified) disulfide partner. The flexible regions are coloured in light blue and fork-like branches depict predicted high-mannose *N*-linked glycans. The β-hairpin region shown to interact with PDI is coloured orange.

In the ER (endoplasmic reticulum), optimal redox conditions are maintained to facilitate formation of native disulfide bonds in secretory proteins. In mammalian cells, disulfide bonds are mainly generated by Ero1 (ER oxidoreductin-1) [1,2]. Proteins of the Ero1 family comprise two conserved di-cysteine active sites [3] (Figure 1). The so-called inner active site sits adjacent to a FAD moiety inside a four-helix bundle, whereas the outer active site (containing the two ‘shuttle’ cysteines) is located on a flexible loop region [4,5]. The inner active site is oxidized by molecular oxygen via FAD, which leads to generation of hydrogen peroxide [6–8]. In turn, the inner active site oxidizes the shuttle cysteines by thiol–disulfide exchange [9]. The shuttle cysteines then oxidize active-site cysteines in members of the PDI (protein disulfide-isomerase) family [2,8,10–12]. As the final step in the Ero1–PDI disulfide relay, PDIs introduce disulfide bonds into newly synthesized proteins in the ER [13].

Two isoforms of Ero1 have been identified in nearly all vertebrates studied so far [14] including humans: Ero1α and Ero1β [15,16]. Whereas Ero1α is widely expressed, Ero1β is predominantly found in select tissues, such as the pancreas and salivary gland [16,17]. Both Ero1 isoforms are up-regulated by the UPR (unfolded protein response), which is a transcriptional and translational programme that is induced by accumulation of misfolded proteins in the ER (designated ER stress). The UPR seeks to restore ER homoeostasis, for example by decreasing the ER protein load through translational arrest and in parallel up-regulating chaperones to assist folding [18]. PERK [PKR (double-stranded-RNA-dependent protein kinase)-like endoplasmic reticulum kinase], Inositol-requiring enzyme 1α (Ire1α) and ATF6α (activating transcription factor 6α) are the three transmembrane transducers that initiate ER-to-nucleus signalling upon ER stress. Whereas the PERK pathway is involved in up-regulating Ero1α [19], Ero1β is induced by the XBP1s transcription factor (which is activated by Ire1α) [20] and ATF6α [21].

Apart from the first three cysteines in Ero1α (Cys^35^, Cys^37^ and Cys^46^), the 12 additional cysteines are conserved in the vertebrate branch of the Ero1 family [22,23]. In addition to these 12 cysteines, human Ero1β contains a cysteine residue in position 262 (Cys^262^) (Figure 1). The disulfide pattern in Ero1α has been mapped by mass spectrometry [23,24] and crystallography [4] (Figure 1). In contrast to Ero1 from *Saccharomyces cerevisiae* (Ero1p) [5], the Ero1α shuttle cysteines (Cys^94^ and Cys^99^) can engage in regulatory disulfide bonds with non-active-site cysteines (Cys^131^ and Cys^104^, respectively) [23,25]. The presence of these two disulfide bonds blocks the outer active site and thus inhibits the activity of Ero1α [23,25]. In the cell, formation and reduction of these inhibitory disulfide bonds depend on the redox state of PDI [23]. This gives rise to a tightly regulated homoeostatic feedback mechanism where Ero1α is only active when oxidized PDI is scarce [23]. Since the ER glutathione redox buffer influences the redox state of PDI [26,27], Ero1α activity is modulated by the ratio between oxidized and reduced glutathione mediated through PDI [28]. Similarly, redox regulation of Ero1p in *S. cerevisiae* is also influenced by the redox state of PDI and glutathione [29–31]. Moreover, the ratio between oxidized and reduced glutathione is tightly balanced in the ER in human cells, which is at least in part a consequence of the Ero1 feedback regulation [2].

In comparison with Ero1α, Ero1β activity does not seem to be as tightly regulated. Whereas overexpressed Ero1α-WT (wild-type Ero1α) is predominantly inactive and therefore has a subtle effect on the redox state of the PDI homologue ERp57 [23,24], overexpression of Ero1β-WT hyperoxidizes ERp57, i.e. leads to a larger fraction of the molecules with active-site cysteines in the disulfide-bonded state [23,32]. Similarly, Ero1β-WT is more active than Ero1α-WT in an *in vitro* oxidation assay performed with PDI as the substrate [7]. On non-reducing SDS–PAGE gels exogenous Ero1β expressed in mammalian cells migrates as two distinct redox species, with the distribution between the faster migrating (OX) and slower migrating species (Red) varying between experiments [23,33,34]. Similar to Ero1α [25], an initial shift from the OX to the Red species of Ero1β was observed during the catalysis of thioredoxin oxidation *in vitro* [7]. When thioredoxin was completely oxidized, the redox state of Ero1β reverted to the OX species [7]. Thus, Ero1β activity is also regulated by intramolecular disulfides.

In Ero1α, a cysteine-to-alanine mutant of Cys^104^ and Cys^131^ (Ero1α-C104A/C131A) displays hyperactivity since it can no longer form the two regulatory disulfides, but retains the two residues of the outer active site, Cys^94^ and Cys^99^ [24,25]. Recently, we showed that overexpression in human cells of the equivalent Ero1β mutant (Ero1β-C100A/C130A) gave rise to more pronounced hyperoxidation of ERp57 relative to overexpression of Ero1β-WT [32], suggesting that the regulatory mechanism is shared for Ero1α and Ero1β. However, Ero1β contains an additional cysteine residue (Cys^262^), which is not present in Ero1α. A disulfide bond between Cys^100^ and Cys^262^ was recently proposed to be present in Ero1β purified from *Escherichia coli* [7]. Moreover, Ero1β-C100A displayed slowed oxidation kinetics relative to Ero1β-WT [7], suggesting that the presence of the proposed Cys^100^–Cys^262^ disulfide bond increases Ero1β activity. On this background, we decided to further investigate the interplay between intramolecular disulfide bonds and regulation of activity in Ero1β.

## MATERIALS AND METHODS

### Primers and plasmids

Human Ero1β-myc6his ([16]; a gift from R. Sitia, Milan) cloned into the pcDNA5/FRT/TO vector [23] was used as a template for QuikChange mutagenesis (Stratagene) to introduce Cys-to-Ala mutations. The following primer was used to generate the C262A mutation (only the sense strand is shown): C262A 5′-GA-CTTCATGCTAGCATCAATTTACATCTAGCCGCAAATTAT-CTTTTGG-3′. The C100A and C130A mutations have been described before [32]. All plasmids were sequenced to confirm the correct DNA sequence of the inserts.

### Cell culture

Dox (doxycycline)-inducible Flp-In T-REx HEK-293 (Life Technologies) cell lines were generated and grown as previously described [23]. Ero1β expression was induced for 24 h (unless otherwise stated) using 1 μg/ml Dox (Sigma). For ER stress induction, cells were treated with either 5 μM thapsigargin (Sigma) or 2.5 μg/ml tunicamycin (Sigma) for the indicated time.

### Sample preparation and AMS (4-acetamido-4′-maleimidylstilbene-2,2′-disulfonic acid) modification

Cells were treated with NEM (*N*-ethylmaleimide) and subsequently lysed as described elsewhere [35]. The AMS (Life Technologies) modification protocol has been described previously [35]. Reduced and oxidized control lysates were obtained from cells treated with 10 mM DTT (dithiothreitol) or 5 mM diamide (both Sigma) for 5 min at 37°C in full growth medium.

### Antibodies and Western blotting

The following mouse monoclonal antibodies were used: αHis (Tetra-His, Qiagen), αmyc (9E10, Covance), αβ-actin (AC-15, Sigma). The rabbit polyclonal antisera used were: αBiP (G8918, Sigma), αERp57 (a gift from A. Helenius, Zürich, Switzerland), αHERP (a gift from L. Hendershot, Memphis, TN, U.S.A.). Western blotting was performed as previously described [24]. The shown Western blots are representative of at least two independent experiments.

### Redox state analysis of Ero1β by TCA (trichloroacetic acid) precipitation and alkylation of free thiols

Cells cultivated to 60–80% confluency in 6 cm dishes were washed twice in PBS. They were then concomitantly lysed and precipitated by incubation in 10% (v/v) TCA for 15 min on ice. Cells were transferred to an Eppendorf tube, centrifuged (16100 ***g***, 4°C, 15 min) and the supernatant was discarded. Pellets were washed once in ice-cold acetone, centrifuged (16100 ***g***, 4°C, 15 min) and resuspended in 100 μl 100 mM Tris–HCl pH 7.0, 8% (v/v) glycerol, 2% (w/v) SDS, 10% dimethyl sulfoxide, 0.01% (w/v) bromocresol purple and 20 mM NEM. Samples were neutralized by drop-wise addition of 1 M Tris–HCl, pH 7.5, 2% SDS until samples turned purple (bromocresol purple changes colour between pH 5.2 and 6.8). The pellets were subsequently dissolved by sonication, incubated at RT in the dark for 1 h and the redox state of Ero1β was determined by non-reducing Western blotting.

## RESULTS

### Structure homology modelling of Ero1β predicts Cys^262^ to be buried in the structure

**Figure 2:**
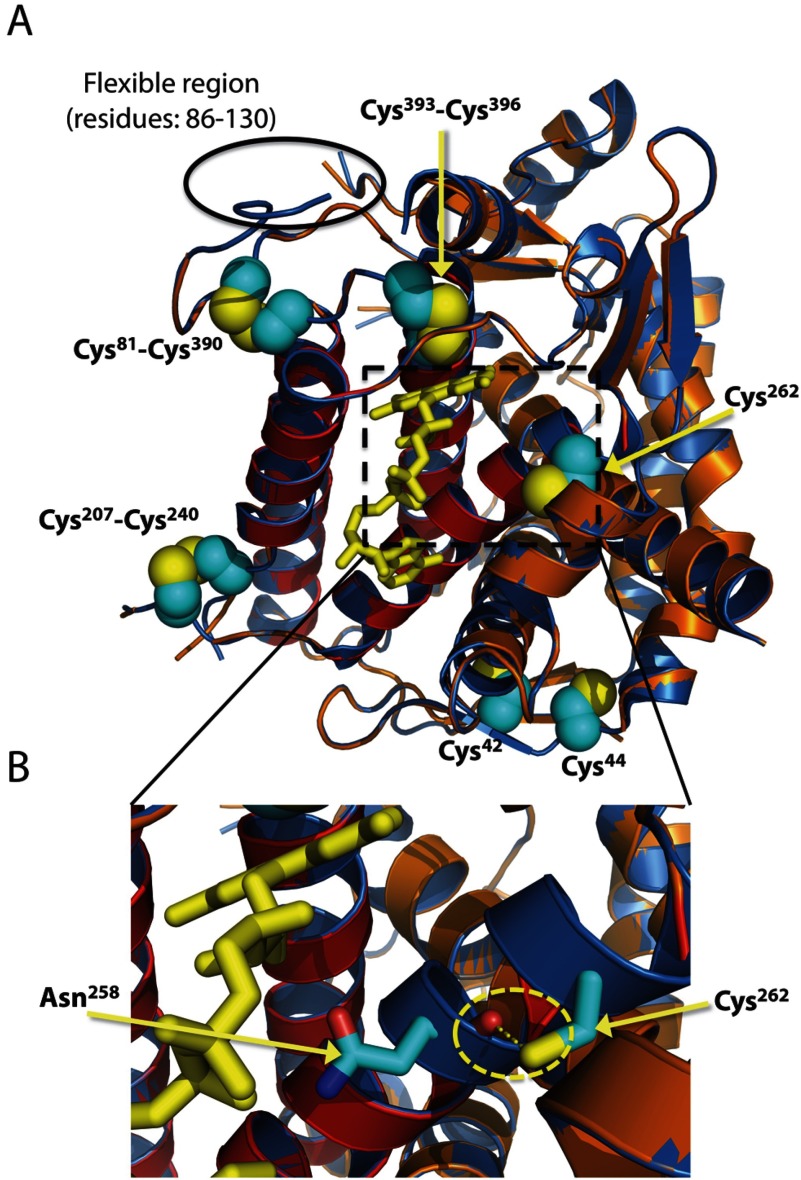
Structural model of Ero1β (**A**) Superimposition of the structure of inactive Ero1α (PDB ID: 3ahr; [4]) and a structural model of Ero1β. The latter was homology modelled based on the structure of inactive Ero1α using the SWISS-MODEL software [36]. Predicted flexible regions (see Figure 1) are not depicted. The approximate position of the flexible region comprising residues 86–130 in Ero1β is indicated. In Ero1β, the α-helices of the four-helix bundle are shown in red, the remainder of the molecule in orange and the cysteine residue side chains are depicted as spheres (yellow: sulfur, cyan: carbon) with amino acid numbering indicated. Ero1α is depicted in blue and the FAD moiety (stick model) in yellow. (**B**) Zoom of the structural overlay in (A). The predicted hydrogen bond (encircled) between the Ero1β Cys^262^ SH-group and the carbonyl O (red) of Asn^258^ is shown by the broken yellow line. In the model, the distance between the S and O atoms is 2.74 Å. This figure was created in PyMOL (http://www.pymol.org).

The amino acid sequences of Ero1β are highly conserved between orthologues (Supplementary Figure S1 available at http://www.bioscirep.org/bsr/034/bsr034e103add.htm). Thus, potential roles of cysteine residues in regulatory disulfide bonds based on evolutionary conservation could not be inferred from a multiple sequence alignment. Instead, we used structure homology modelling of Ero1β to assess the proposed disulfide patterns in the protein (Figures 1A and 1B). The protein structure prediction software SWISS-MODEL [36] was used to predict the three-dimensional structure of Ero1β based on the crystal structure of inactive Ero1α, a mutant in essence corresponding to the OX2 form ([4]; PDB ID: 3AHR) (Figure 2A). The sequences of mature Ero1α and Ero1β are highly similar [14] with a sequence identity of 65%. As expected from the high sequence conservation, the α-helical fold in Ero1α was predicted to be preserved in Ero1β including the four-helix bundle involved in FAD binding (Figure 2A, red-coloured α-helices). The structure of the flexible region (residues 86–130) comprising the proposed Cys^90^–Cys^130^ or the Cys^95^-Cys^100^ disulfide bonds could not be reliably modelled (Figure 2A).

In contrast to the cysteines in the flexible region, Cys^262^ is located at the end of a conserved helix [14], which is part of the four-helix bundle (Figure 2A). Moreover, Cys^262^ is positioned close to a protruding β-hairpin, which is critical for the interaction with PDI [37]. The equivalent residue in Ero1α (Ser^263^) is completely buried. Similarly, in the Ero1β model, Cys^262^ is predicted to have a relative accessible surface area of 0 (as calculated by the ASAView software [38] and the GETAREA method [39]), which strongly suggests that Cys^262^ in Ero1β is buried in the native structure. Moreover, the side chain −SH (Cys^262^)/−OH (Ser^263^) is predicted to form a hydrogen bond with the backbone carbonyl group of Asn^258^/Asn^259^, respectively (Figure 2B). This hydrogen bond seems to be part of a conserved hydrogen bond network, including hydrogen bonds from the side chain of Asn^258^/Asn^259^ to FAD, which helps stabilize the structure in the vicinity of the bound cofactor. Finally, we also note that in *Xenopus tropicalis* Ero1β, a serine residue is found in place of Cys^262^ (Supplementary Figure S1), indicating that a cysteine is not strictly necessary at this position as may have been expected if it played an important function in regulating the activity of the enzyme.

### SDS–PAGE mobility of Ero1β mutants suggests conservation of regulatory disulfide bonds in Ero1α and Ero1β

**Figure 3:**
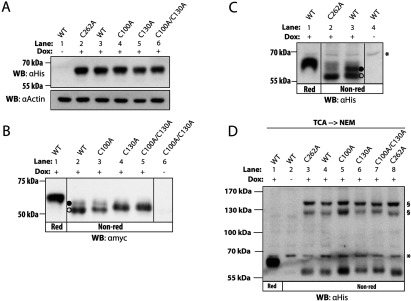
SDS–PAGE mobility of Ero1β variants suggests that Ero1α and Ero1β share their sets of regulatory disulfide bonds (**A**) Expression of His- and Myc-tagged Ero1β variants was induced with Dox for 24 h and cells were NEM treated to alkylate-free thiols. Equal amounts of protein from lysates were analysed by reducing SDS–PAGE and Western blotting using αHis (Ero1β) and αActin (loading control) to compare expression levels of Ero1β variants. (**B**,**C**) Cell lysates were obtained as in (**A**). The SDS–PAGE mobility of the Ero1β variants was analysed under non-reducing (Non-red) or reducing (Red) conditions by αmyc or αHis Western blotting. The open and filled circles indicate the previously described OX and Red redox forms of Ero1β WT [33], respectively, and vertical hairlines denote removal of lanes. Asterisk denotes a background band. (**D**) Expression of Ero1β variants was induced as in (**A**). Cells were subjected to TCA precipitation to rapidly quench thiol–disulfide exchange reactions and to denature cellular proteins. Precipitates were redissolved in a buffer containing NEM to alkylate free thiols. Subsequently, the SDS–PAGE mobility of the Ero1β variants was analysed under non-reducing conditions by αHis Western blotting. Section signs (§) indicate possible Ero1β mixed-disulfide dimeric species and the asterisk (*) denotes a background band.

To investigate the structural importance of intramolecular disulfide bonds in human Ero1β, we expressed Ero1β cysteine mutants in human cells and analysed the mobility of these mutants by non-reducing SDS–PAGE. Apart from already established stable cell lines for ectopic inducible expression of Ero1β-WT [23] and Ero1β–C100A/C130A [32], we generated three new inducible cell lines for the following mutants: Ero1β-C100A, Ero1β–C130A and Ero1β–C262A. As compared with Ero1β-WT, Ero1β–C100A and Ero1β–C262A showed similar expression levels, whereas the expression levels of Ero1β–C130A and Ero1β–C100/130A were lower (Figure 3A). Importantly, none of the cell lines overexpressing Ero1β mutants of Cys^100^ and/or Cys^130^, which turned out to be hyperactive (see below), expressed more protein than the Ero1β-WT-expressing cell line.

As previously observed [33], the monomeric form of exogenous Ero1β-WT migrated as two distinct redox species (Red and OX) when cells were treated with NEM to alkylate free thiols *in situ* prior to lysis (Figure 3B, lane 2 and Figure 3C, lane 3). However, upon TCA precipitation with subsequent NEM treatment, monomeric Ero1β-WT migrated as one redox species (Figure 3D, lane 4). TCA precipitation rapidly quenches thiol–disulfide exchange reactions and denatures proteins, enabling alkylation of thiols buried in the native structure [40]. When cells are *in situ* NEM-treated, approximately 20% of the cellular protein thiols have been shown to be inaccessible to NEM [41]. Such NEM inaccessibility is thought to be a consequence of these thiols being buried in the native structure [40]. We therefore suggest that inefficient alkylation of (a) free thiol(s) buried in the structure of Ero1β gives rise to rearrangement of disulfide bonds upon denaturation, leading to the appearance of the Red Ero1β redox form (Figures 3B and 3C). Conversely, when all free thiols are efficiently alkylated, Ero1β-WT is preserved as a single redox species visible on SDS–PAGE gels (Figure 3D).

The SDS–PAGE mobility of the Ero1β variants on non-reducing gels (Figure 3D) is consistent with Ero1β having a similar pattern of disulfide bonds as Ero1α (Figures 1B and 1C). We were able to detect a relatively small migration shift between Ero1β-WT and Ero1β-C100A (Figure 3D, lanes 4–5) suggesting that Cys^100^ is not engaged in a long-range disulfide bond. In contrast, a larger shift was observed upon mutation of Cys^130^ (Figure 3D, lanes 6–7) consistent with removal of the longer-ranging Cys^90^–Cys^130^ disulfide bond. No redox species of Ero1β-C100A co-migrated with Ero1β–C130A (Figure 3D, lanes 5–6), suggesting that the Cys^90^–Cys^130^ disulfide bond is intact in Ero1β–C100A.

A fraction of Ero1β is present as a disulfide-bonded homo-dimer in human cells [33] and when expressed in bacteria [7]. Moreover, Ero1β engages in heterodimeric mixed-disulfide species with PDI and ERp44 in human cells [42]. The possible dimeric species involving Ero1β-WT and Ero1β–C262A were similar (Figure 3D, lanes 3–4), suggesting that Cys^262^ is not involved in formation of mixed-disulfide dimeric species. Notably, Ero1β–C262A did not migrate slower than Ero1β-WT (Figure 3D, lanes 3–4), suggesting that Cys^262^ is not engaged in a long-range disulfide bond. Instead, Ero1β–C262A was present exclusively as the OX redox species in lysates from cells treated *in situ* with NEM (Figure 3C, lane 2), and as a single redox species co-migrating with Ero1β-WT in lysates from cells subjected to TCA precipitation (Figure 3D, lane 3). This clearly suggests that non-native *ex vivo* disulfide shuffling in lysates of *in situ* NEM-treated cells observed for Ero1β-WT (Figures 3B and 3C) depends on the presence of Cys^262^, and that this residue is inaccessible to NEM in the native structure.

Based on these results, we propose that the regulatory disulfide bonds in Ero1α (Cys^94^–Cys^131^ and Cys^99^–Cys^104^) are conserved in Ero1β (Cys^90^–Cys^130^ and Cys^95^–Cys^100^) and that Cys^262^ constitutes a poorly accessible free thiol in the native structure. The deduced disulfide pattern in the OX redox form of Ero1β is shown in Figure 1(B).

### Removal of either of the regulatory disulfide bonds increases the activity of Ero1β in cells

**Figure 4:**
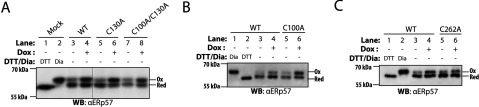
Hyperoxidation of ERp57 is intensified by removal of regulatory disulfide bonds in Ero1β (**A–C**) Where indicated, expression of Ero1β variants was induced with Dox for 24 h. Prior to lysis, cells were treated with NEM to alkylate free thiols. After cell lysis, cysteines present in disulfides were reduced and decorated with AMS. Such AMS modification of active-site cysteines originally present in the oxidized state gives rise to slower SDS–PAGE mobility compared with the (NEM-decorated) pool of ERp57 containing reduced active-site cysteines. The cellular redox state of ERp57 was visualized by Western blotting. DTT and Diamide (Dia) treated-cells were used to show the mobility of fully oxidized (Ox) and reduced (Red) ERp57. A vertical hairline denotes removal of lanes.

We next wanted to assess the relative contribution of the proposed disulfide bonds (Cys^90^–Cys^130^ and Cys^95^–Cys^100^) to the regulation of Ero1β activity. First, we analysed the cellular redox state of ERp57, as assessed by differential alkylation of the active-site cysteines. This assay probes the ratio of ERp57 molecules with active-site cysteines in the oxidized and reduced state, respectively, and has been used routinely in the field as readout for changes in the ER redox environment [23,27,43]. Mutation of Cys^100^ and Cys^130^ alone or in combination increased the hyperoxidizing effect of Ero1β on the redox state of ERp57 relative to Ero1β-WT (Figures 4A and 4B). This suggests that both disulfide bonds (Cys^90^–Cys^130^ and Cys^95^–Cys^100^) are involved in inhibiting the activity of Ero1β. Consistent with Cys^262^ not being involved in regulation of Ero1β activity, overexpression of Ero1β–C262A showed only a minor hyperoxidizing effect on ERp57 (Figure 4C). It should be noted that consistent with our previous studies, and as noted before [2], the ERp57 redox state differed between individual experiments likely reflecting physiological variations. This, however, does not affect the overall conclusions concerning the relative oxidizing effects of overexpressing different Ero1β variants.

**Figure 5:**
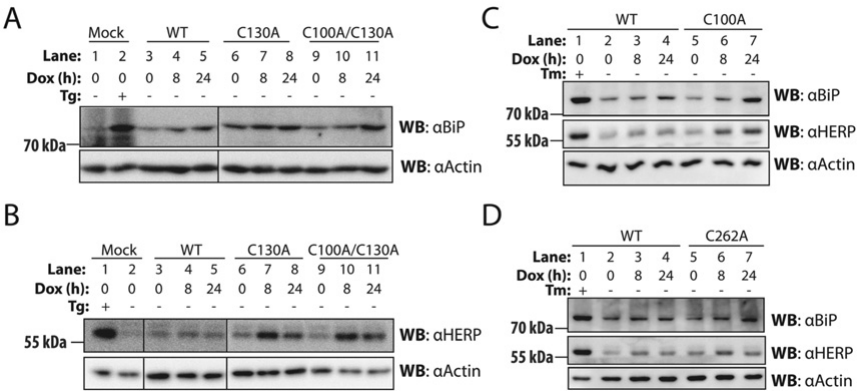
Absence of regulatory disulfide bonds in Ero1β increases induction of the unfolded protein response (UPR) (**A–D**) Expression of Ero1β variants was induced with doxycycline (Dox) for the indicated periods of time. Expression levels of BiP and HERP were analysed by Western blotting using αActin as loading control. The mock cell line is stably transfected with an empty vector. Cells were either treated with 5 μM thapsigargin (Tg) for 18 h (**A**) and 6 h (**B**) or treated with 2.5 μg/ml tunicamycin (Tm) for 20 h (**C**, **D**) to generate positive control lysates for induction of the UPR.

We recently showed that a deregulated Ero1α mutant (Ero1α-C104A/C131A) markedly activated the UPR as a result of its increased oxidase activity when overexpressed in HEK-293 cells (human embryonic kidney cells) [24]. To study whether overexpression of Ero1β also induces the UPR, we analysed the protein levels of the two established UPR targets, BiP (immunoglobulin heavy-chain-binding protein) and HERP (homocysteine-induced ER protein) [44]. As expected from the loose regulation of Ero1β (Figure 4), BiP and HERP levels were moderately increased upon expression of Ero1β-WT (Figures 5A–5D). These effects were more pronounced upon mutation of Cys^100^ and Cys^130^ alone or in combination (Figures 5A–5C), correlating with the impact of these mutants on the redox state of ERp57 (Figures 4A and 4B). Overexpression of Ero1β-C262A showed a similar degree of UPR induction as Ero1β-WT (Figure 5D). These findings suggested that Cys^262^ is not involved in regulating Ero1β activity, and is in keeping with our proposed disulfide pattern of Ero1β (Figure 1B).

## DISCUSSION

Tight regulation of Ero1α activity is important to maintain balanced ER redox conditions [23–25]. We propose that the regulatory disulfide bonds in Ero1α and Ero1β are conserved (Figures 1B and 1C). This conclusion is based on several lines of evidence, including molecular modelling of the Ero1β structure (Figure 2), SDS–PAGE mobility analysis of Ero1β mutants (Figures 3B–3D) and ER redox (Figure 4) and ER stress readouts (Figure 5). Overall, the findings that overexpression of Ero1β mutants devoid of Cys^100^ and/or Cys^130^ induces the UPR, hyperoxidizes ERp57 and that Ero1β-C100A/C130A hyperoxidizes an ER-localized glutathione sensor [32], indicate that the underlying mechanism is likely to involve an oxidizing perturbation of the ER redox environment, which in turn results in protein misfolding and therefore activation of the UPR.

In a previous study [7], Ero1β-C262A purified from *E. coli* displayed a prominent slow-migrating redox species when compared with Ero1β-WT by non-reducing SDS–PAGE, indicating the loss of a long-range disulfide. Furthermore, analysis of tryptic fragments supported the presence of a Cys^100^–Cys^262^ disulfide bond. Finally, the Ero1β-C100A mutant was less active *in vitro* than Ero1β-WT, suggesting that the presence of the proposed Cys^100^–Cys^262^ disulfide bond positively regulates the activity of Ero1β.

Here, we expressed Ero1β (and mutants thereof) in its native environment in the ER of human cells and reached the conclusion that a disulfide bond between Cys^100^ and Cys^262^ is not likely to form. Thus, mutation of Cys^100^ rendered Ero1β hyperactive and overexpression of Ero1β-C262A showed effects comparable to Ero1β-WT overexpression. We also provide two-fold evidence that Cys^262^ is a solvent inaccessible residue in the native structure of Ero1β. First, a fraction of Ero1β-WT molecules rearrange into a redox species that migrates as the Red form upon *in situ* NEM treatment in a Cys^262^-dependent manner, suggesting that NEM cannot gain access to Cys^262^ under native conditions. Secondly, a homology model of Ero1β based on the crystal structure of Ero1α places Cys^262^ in a non-solvent exposed site in a highly conserved α-helix. Collectively, these findings strongly support the conclusion that Cys^262^ does not engage in an intramolecular disulfide bond with Cys^100^. To verify the proposed disulfide pattern, we also sought to map the intramolecular disulfides in Ero1β purified from human cells by mass spectrometry, as has previously been achieved for Ero1α [23]. Unfortunately, the results obtained by this approach were ambiguous (H. G. Hansen, L. Ellgaard and F. Hubálek, unpublished work), which was likely a result of disulfide bond scrambling in the course of sample preparation.

Using TCA precipitation and subsequent NEM treatment, we demonstrated that the Red form of Ero1β [33] is likely an artefact of inefficient thiol alkylation, indicating that overexpressed Ero1β is present solely as the OX redox species. Unfortunately, the redox state of endogenous Ero1β assessed by SDS–PAGE mobility under non-reducing conditions is currently unknown. Moreover, we currently do not know why Ero1β migrates 5–7 kDa faster than Ero1α on non-reducing SDS–PAGE gels [23], even though the predicted molecular mass of mature Ero1β is only 1–2 kDa smaller than the corresponding mass of mature Ero1α. Since deglycosylation of Ero1β gives rise to a more pronounced mobility shift on SDS–PAGE gels as compared with Ero1α [16], the presence of N-linked glycans cannot explain the unexpectedly large difference in SDS–PAGE mobility between Ero1α and Ero1β.

As Ero1α and Ero1β likely share their sets of regulatory disulfide bonds, features other than a distinct pattern of disulfide bonds must determine the loose redox regulation of Ero1β relative to Ero1α. Mutation of the Cys^394^–Phe–Lys–Cys^397^ inner active site sequence of Ero1α to the Ero1β sequence (Cys^393^–Asp–Lys–Cys^396^) substantially increases the oxidase activity of Ero1α [7]. This suggests that Asp^394^ in Ero1β contributes to the apparently loose redox regulation of Ero1β relative to Ero1α.

As previously proposed [22], the loose regulation of Ero1β activity relative to Ero1α could be explained by a higher reduction potential of the regulatory disulfide bonds in Ero1β. The high expression of Ero1β in the pancreas and salivary gland indicates a specific role of the protein in secretory tissues. Accordingly, oxidative folding of pro-insulin is impeded in pancreatic islet cells derived from Ero1β-compromised mice, an effect that is not exacerbated by concomitantly compromising Ero1α function [45]. However, increasing disulfide-bond formation by exogenous Ero1α expression stimulates oxidative folding of pro-insulin [46]. These observations suggest that the loose regulation of Ero1β activity could have evolved to optimally support the high demand of disulfide bonds in secretory tissues.

## Online data

Supplementary data
