# Supplementary material for: Biochemical evidence that regulation of Ero1β activity in human cells does not involve the isoform-specific cysteine 262
Source: Biosci Rep. 2014 Mar 31;34(2):e00103. doi: 10.1042/BSR20130124 (PMC3971451; doi:10.1042/BSR20130124)
Supplement: Supplementary data [file bsr034e103add.pdf]

Biosci. Rep. (2014) / **34** / art:e00103 / doi 10.1042/BSR20130124

OPEN ACCESS

Original Paper

SUPPLEMENTARY DATA

Biochemical evidence that regulation of Ero1β activity in human cells does not involve the isoform-specific cysteine 262

Henning G. HANSEN*[1], Cecilie L. SØLTOFT*, Jonas D. SCHMIDT*[2], Julia BIRK†, Christian APPENZELLER-HERZOG*† and Lars ELLGAARD*[3]

*Department of Biology, University of Copenhagen, 2200 Copenhagen, Denmark
†Department of Pharmaceutical Sciences, University of Basel, 4056 Basel, Switzerland

Supplementary Figure S1 is on the following page.

Bioscience Reports www.bioscirep.org

[1] Present address: Novo Nordisk Foundation Center for Biosustainability, Technical University of Denmark, 2970 Hørsholm, Denmark.
[2] Present address: Department of International Health, Immunology and Microbiology, University of Copenhagen, 2200 Copenhagen, Denmark.
[3] To whom correspondence should be addressed (email: lellgaard@bio.ku.dk).

© 2014 The Author(s) This is an Open Access article distributed under the terms of the Creative Commons Attribution Licence (CC-BY) (http://creativecommons.org/licenses/by/3.0/) which permits unrestricted use, distribution and reproduction in any medium, provided the original work is properly cited.

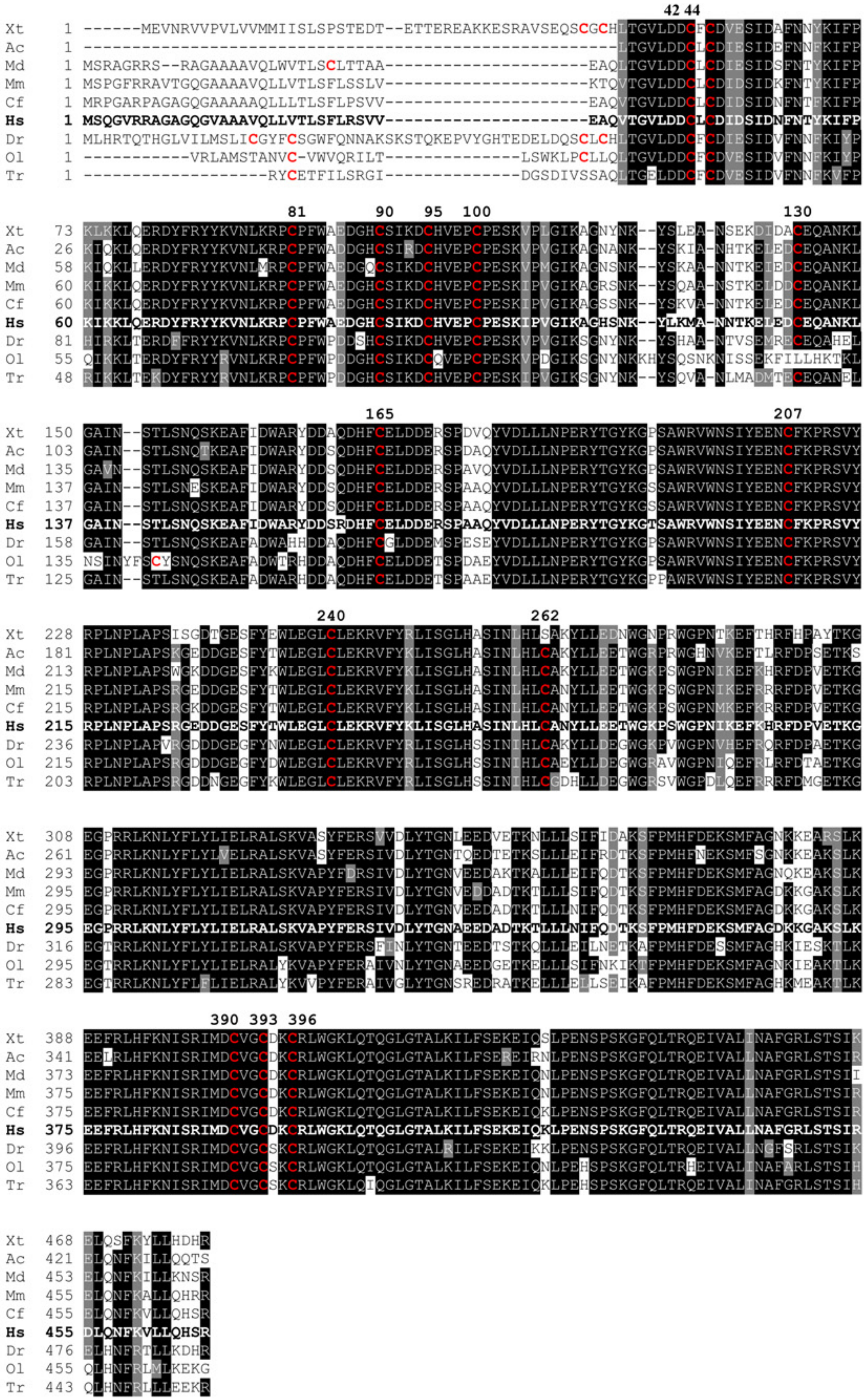

© 2014 The Author(s) This is an Open Access article distributed under the terms of the Creative Commons Attribution Licence (CC-BY) (http://creativecommons.org/licenses/by/3.0/) which permits unrestricted use, distribution and reproduction in any medium, provided the original work is properly cited.

Figure S1 Evolutionary conservation of Ero1β
A multiple sequence alignment of Ero1β orthologs was performed with Muscle [1] using the following UniProt entries. *Xenopus tropicalis* (frog; F6ULN4); *Gallus gallus* (chicken; E1C917); *Anolis carolinensis* (lizard; G1KAL4); *Monodelphis domestica* (opossum; F7CL82); *Mus musculus* (mouse: Q8R2E9); *Canis familiaris* (dog; F1Q091); *Homo sapiens* (human; Q86YB8); *Danio rerio* (zebrafish; E7F2A8); *Oryzias latipes* (rice fish; H2L719); *Takifugu rubripes* (pufferfish; H2TT03). Black boxes indicate amino acid identities and grey boxes show amino acid similarities when found in at least seven of the nine sequences. The human sequence is shown in boldface, cysteine residues are shown in red colour and amino acid position of the cysteine residues in the human sequence is indicated above the alignment.

REFERENCE

1 Edgar, R. C. (2004) MUSCLE: multiple sequence alignment with high accuracy and high throughput. Nucleic Acids Res. **32**, 1792–1797

Received 19 November 2013/8 January 2014; accepted 28 January 2014

Published as Immediate Publication 25 February 2014, doi 10.1042/BSR20130124

© 2014 The Author(s) This is an Open Access article distributed under the terms of the Creative Commons Attribution Licence (CC-BY) (http://creativecommons.org/licenses/by/3.0/) which permits unrestricted use, distribution and reproduction in any medium, provided the original work is properly cited.
